# X-ray free Percutaneous Nephrolithotomy (PCNL) with Alken Telescopic Metal Dilator for large stone in horseshoe kidney: A case report

**DOI:** 10.1016/j.eucr.2023.102616

**Published:** 2023-11-15

**Authors:** Rinaldo Indra Rachman, Nur Rasyid, Ghifari Nurullah, Widi Atmoko, Sahat Matondang, Ponco Birowo

**Affiliations:** a Department of Urology, Faculty of Medicine, Universitas Indonesia, Cipto Mangunkusumo Hospital, Jalan Diponegoro No. 71, Jakarta, 10430, Indonesia; b Department of Radiology, Cipto Mangunkusumo Hospital, Jalan Diponegoro No. 71, Jakarta, 10430, Indonesia

**Keywords:** X-ray free, PCNL, ATMD, Stone, Horseshoe kidney

## Abstract

Percutaneous Nephrolithotomy (PCNL) has been shown as a safe and effective method for treating nephrolithiasis in horseshoe kidney patients. We report the first X-ray Free PCNL with Alken Telescopic Metal Dilator (ATMD) in horseshoe kidney.

A 58-Year-Old female was diagnosed with left large staghorn stone and horseshoe kidney treated with X-ray Free PCNL with ATMD.

X-ray Free PCNL with ATMD is safe and effective nephrolithiasis lithotripsy for staghorn stone in Horseshoe Kidney. This technique is the first of its kind and possibly has become an excellent alternative for urological centers without access to fluoroscopy.

## Highlights

• X-ray free PCNL is a novel surgical technique which is very safe for surgeons and patients alike.
• Our center performed the first case of treating large stone in horseshoe kidney with X-ray free PCNL.
• This technique is feasible to be performed in treating large stone in horseshoe kidney.
• ATMD is the most cost-effective dilator method in PCNL, which is very suitable for developing countries.

## 1 Introduction

Horseshoe kidney is one of the most common congenital renal fusion anomalies.^1^ It is also associated with syndromes and has a higher risk for future morbidities such as renal stone formation and malignancy.^1^ Previous studies have also shown that PCNL is a safe modality to treat renal stones in Horseshoe Kidney with a high success rate of up to 90.5–92 %.^1^ Retrograde access may be challenging to perform in horseshoe kidney patients, as the abnormal collecting system resulting from malrotation of the kidney can further complicate the procedure.^2^ To the authors' knowledge, this is the first case of a large stone in a horseshoe kidney patient treated with X-Ray free PCNL with ATMD.

## 2 Case presentation

**Fig. 1:**
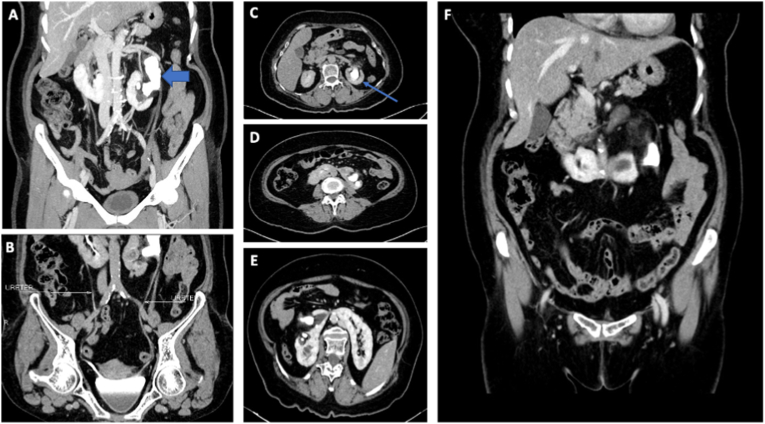
(A) Preoperative CT-Scan showing staghorn stone in left kidney, (B) Preoperative CT-Scan showing normal ureter, (C) Puncture site (superior pole) (D–F) Preoperative CT-Scan showing horseshoe Kidney.

A 58-year-old female was referred to our urology clinic from a district hospital with dull left flank pain three months ago. Non-Contrast CT showed horseshoe kidney with left staghorn stone 5.4 cm × 1.3 cm x 3.1 cm (HU 703–830) in major superior-inferior calyx to the proximal left ureter and several oval-shaped stones with grade III-IV left hydronephrosis Fig. 1A. Preoperative ureter evaluation is essential to ensure that the patient's left ureter is normal for procedure planning. It is a prerequisite examination for X-Ray Free PCNL with ATMD. This patient had a normal left ureter Fig. 1B. Fig. 1C showed clear visualization of the staghorn stone and the puncture site (superior pole), whereas Fig. 1D-F showed horseshoe kidney.

**Fig. 2:**
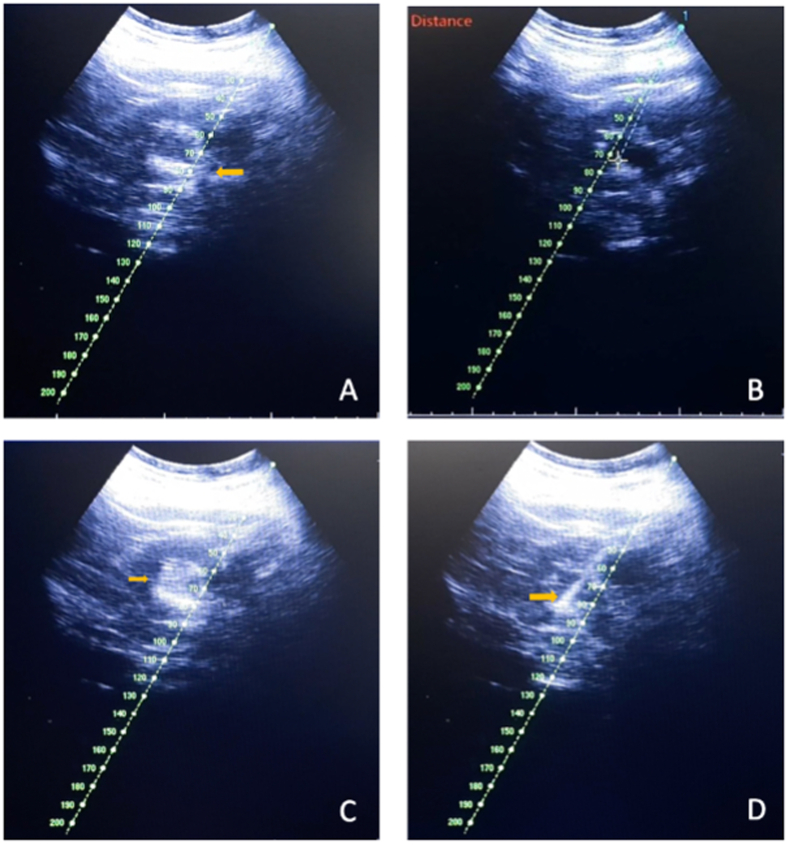
(A) Ureteral Catheter Visualization in US Examination, (B) Skin to stone distance in US Examination, (C) Water-Jet Appearance in US Examination, (D) Visualization of ATMD through US Examination.

Totally X-ray free PCNL was performed to the patient. The patient was positioned in modified-supine position. Ureteral catheter placement in the collecting system was confirmed by the visualization of the ureteral catheter using ultrasound during insertion Fig. 2A. Skin-to-stone distance was measured 70.86 mm Fig. 2B. Water-jet appearance on ultrasound during normal saline flushing from the ureteral catheter was seen in Fig. 2C.

Normal saline was pumped into the collecting system via the ureteral catheter to create artificial hydronephrosis. A thorough Ultrasound examination (Terason Portable Digital Diagnostic Ultrasound Machine) evaluation of the kidney and nearby structures was performed including ensuring there was no retro-renal colon.

Needle calibration was performed before puncture. Puncture was then performed using a 17.5 G, 22 cm puncture needle guided by ultrasound to the patient's superior calyx Fig. 1C. The time to puncture (from initial kidney ultrasound imaging to successful puncture) was 1 minute 54 seconds. The presence of urine flowing from the puncture needle confirms a successful puncture.

A 0.035-inch J-shaped stiff guidewire was inserted through the puncture needle. After that, fascial dilatation under ultrasound guidance was performed using a fascial dilator 8 Fr up to 12 Fr. Tract dilatation was performed using ATMD increasing in size from 9 Fr gradually up to 24 Fr Fig. 2D. Thereafter inner and outer Amplatz sheath was inserted under ultrasound guidance. The dilation time took 8 minutes and 17 seconds. All steps were guided using US.

**Fig. 3:**
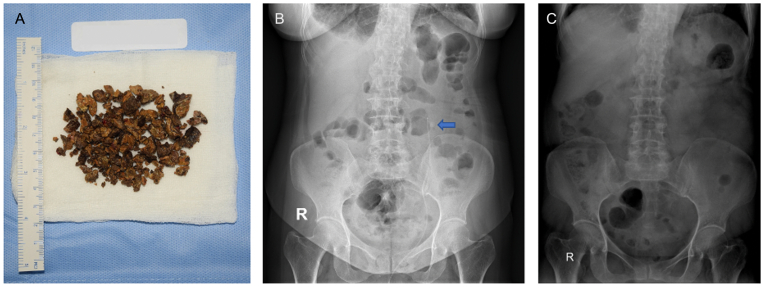
(A) Evacuated stone fragments, (B) postoperative kidney-ureter-Bladder X-Ray, (C) Post ESWL KUB X-ray.

A 25 Fr rigid nephroscope was inserted and stone was fragmented with a combination of a 6 Fr pneumatic lithotripter and an 11.3 Fr shock pulse lithotripter. The evacuated stone fragments are shown in Fig. 3A.

There was no notable complication noted during this procedure. Postoperative KUB X-ray showed an 8 mm residual stone with intact ureteral catheter (Fig. 3B). The patient was then discharged on day three postoperative. She was then treated with extracorporeal shockwave lithotripsy (ESWL) for two sessions. KUB X-ray showed a decreased stone size from 0.8 cm to 0.5 cm. (Fig. 3B & C). The patient was asymptomatic at the end of the treatment.

## 3 Discussion

The main drawback of X-ray imaging is radiation hazard, both to the operators and patients.^3^ There was no statistical difference in terms of efficacy and safety found between fluoroscopy-guided and ultrasound-guided PCNL. Therefore, ultrasound-guided PCNL is a safer and feasible operative method. However it requires a competent operator.^3^ In this case, no significant complications occurred during and after the treatment. Identical to our report, several meta-analyses and systematic reviews also show no statistical difference between US to fluoroscopy guided PCNL. Howevera a study by Yang et al. showed a statistically lower complication rate in US guided PCNL compared to fluoroscopy guided.^4^

X-ray-free ultrasound-guided PCNL procedures mostly use balloon catheter dilators for tract dilatation. In our case, the dilator used was ATMD for access dilatation since these are reusable and can provide a tamponade effect along the dilatation process and become a more cost-effective alternative.^5^ Moreover the unit cost for baloon dilator is around $600, whereas the unit cost for using ATMD is only $1.25. As compared to amplatz dilators, tract formation time with ATMD was shorter (6.56–3.04 vs 5.42–3.07 min, P < 0.001), moreover it is more cost effective with the approximate cost for amplatz dilator were $220 per use.^6^

In this case, we performed a single superior posterior calyx puncture. In a horseshoe kidney, the kidney is positioned more caudally. Previous studies have also shown that the most preferred access site to horseshoe kidneys is the superior calyx (66.64 %), followed by lower calyx (19.04 %) and middle calyx (9.52 %).^5^ More caudal position of horseshoe kidneys may minimize the risk of pleural injury.

Total X-ray free PCNL is a novel technique developed in our center. It is preferred than traditional PCNL given its advantage of 0 radiation hazard. The requirement that must be met for this technique is that the patient must have a normal ureter in the ipsilateral side of the stone. Therefore, pre-procedure ureteral reconstruction is mandatory to evaluate this matter. The operator must be fluent in performing traditional PCNL before performing total X-Ray free PCNL. Avoiding colon perforation is an essential aspect of PCNL procedure, especially in horseshoe kidney patients. In horseshoe kidney, the developmental defect will cause retro-renal displacement of the colon, especially in the left kidney. In our case, we could identify nearby structures using US before puncture. Previous studies have shown that age and horseshoe kidney are significant independent risk factors for colonic perforation during PCNL.^5^ This is especially important, as colonic injury can be unrecognized and life-threatening to the patient.

## 4 Conclusion

X-Ray Free PCNL with ATMD in horseshoe kidney is possible and have shown favorable outcome. This technique is the first of its kind and possibly become an excellent alternative with urological centers without access to fluoroscopy.

## Funding

This article is funded by *HIBAH PUTI 2023* 10.13039/501100006378Universitas Indonesia NKB-393/UN2.RST/HKP.05.00/2023.

## Declaration of competing interest

The authors have no conflicts of interest to declare.
